# Using Blood Group Genotyping to Predict Hemolysis in Patients With β-Thalassemia Major With Frequent Transfusions: Protocol for a Cross-Sectional Study

**DOI:** 10.2196/64379

**Published:** 2025-05-30

**Authors:** Vitasari Indriani, Teguh Triyono, Budi Mulyono

**Affiliations:** 1 Department of Clinical Pathology and Laboratory Medicine Faculty of Medicine Jenderal Soedirman University Banyumas Indonesia; 2 Department of Clinical Pathology and Laboratory Medicine Faculty of Medicine Gadjah Mada University Sleman Indonesia

**Keywords:** alloimmunization, phenotype, genotype, hemolysis, thalassemia, blood group, transfusions, study protocol, Indonesia, cross-sectional study, gene sequencing, hemoglobin, blood transfusion therapy

## Abstract

**Background:**

Hemolytic transfusion reactions are a major complication in patients with β-thalassemia major receiving regular transfusions. These reactions can be influenced by blood group incompatibilities, particularly in settings with limited genotyping practices. In Indonesia, the role of blood group genotyping in predicting hemolysis has not been thoroughly studied.

**Objective:**

This study aims to analyze the association between blood group genotyping and the incidence of hemolysis in people with thalassemia undergoing repeated transfusions.

**Methods:**

This is a cross-sectional study involving people with β-thalassemia major younger than 18 years old who received regular transfusion with intervals of 2-4 weeks and have received more than 20 units of transfusion. Participants with leukemia, lymphoproliferative diseases, diabetes, solid tumors, and immunosuppression disorders were excluded from the study. Genotyping examination was conducted using Allele-Specific polymerase chain reaction (PCR ASP) while phenotyping was examined using immunoserology. Follow-up gene sequencing was conducted to observe the blood group variants. Hemolysis was assessed using several markers such as haptoglobin, free hemoglobin, lactate dehydrogenase, bilirubin, and hemoglobinuria, measured by Cobas C113, the enzyme-linked immunosorbent assay, and urinalysis.

**Results:**

Clinical and laboratory data collection is completed. A total of 90 samples were collected, data analyses were undertaken, and the initial results were reported in September 2024.

**Conclusions:**

The results of this study will provide information on the blood groups’ systems that can predict hemolysis occurrence in patients with β-thalassemia undergoing repeated transfusion. These data will contribute to the best possible patient care management and blood transfusion therapy, thereby reducing the risk of hemolysis and improving the quality of life for patients with thalassemia in Indonesia.

**International Registered Report Identifier (IRRID):**

RR1-10.2196/64379

## Introduction

### Background

β-Thalassemia is particularly prevalent in the Mediterranean, Middle East, and Southeast Asia. Annually, over 40,000 babies are born with β-thalassemia, with about 25,500 cases being transfusion-dependent thalassemia. Patients with transfusion-dependent thalassemia require lifelong packed red cell transfusions to maintain hemoglobin levels between 9 and 11.5 g/dL. In Southeast Asia alone, 20,420 newborns are affected each year, with the Mediterranean and Europe seeing 9914 and 1019 cases, respectively, and 341 cases occurring in America. According to the World Health Organization, Indonesia has a β-thalassemia carrier prevalence of 3%-10% per year [1], corroborated by the Ministry of Health of the Republic of Indonesia, which reports that 3.8% of the Indonesian population is carrier [2].

Patients with β-thalassemia who require repeated blood transfusions are at risk of transfusion-related complications, including excessive iron accumulation, alloimmunization, infections, and transfusion reactions [3]. We can divide transfusion reactions into two types: (1) acute transfusion reactions, which can occur from the time of transfusion to 24 hours after transfusion, and (2) delayed transfusion reactions, which can occur more than 24 hours to 1.5 months after transfusion. Delayed hemolytic transfusion reactions are particularly concerning in patients with thalassemia due to the chronic nature of their transfusion requirements. These reactions can lead to significant morbidity, including fever, decreased hemoglobin levels, and elevated markers of hemolysis, such as lactate dehydrogenase (LDH), bilirubin, and free hemoglobin [4]. Free hemoglobin can be measured in the decreased serum haptoglobin and hemoglobinuria due to intravascular hemolysis [5,6].

Lifelong blood transfusions can lead to the development of anti–red blood cell (RBC) antibodies, including alloantibodies and autoantibodies. The emergence of alloantibodies and autoantibodies to RBC antigens will impede the acquisition of compatible crosses, shorten transfused cells’ in vivo survival, increase iron deposition in tissues, and delay the provision of safe transfusions [4]. The homogeneity of donor and recipient populations, RBC phenotype matching policies, and age at transfusion initiation can influence the frequency of alloimmunization, according to research and literature [4,7-9]. The incidence of alloimmunization in thalassemia is reported to range from 4% to 50%, with anti-E, anti-C, and anti-Kell being the most common alloantibodies identified [7,8]. These rates are influenced by factors such as donor-recipient population homogeneity, RBC phenotype matching policies, and age at the start of transfusion [4,7-9].

Current national guidelines in Indonesia for pretransfusion-packed red cell examination primarily focus on ABO and Rhesus D blood group systems. However, additional clinically significant blood group antigens, such as those within the Kell system, are often not routinely matched, which could increase the risk of alloimmunization and subsequent hemolytic reactions [4,10]. Moreover, many transfusions do not use leukoreduced products, which further increases the risk of transfusion reactions.

This study addresses the limited comprehensive assessment of blood group genotyping in Indonesia. The primary aim is to assess the association between blood group genotyping and hemolysis in patients with thalassemia in the Banyumas region of Indonesia, using molecular genotyping data to provide a more refined understanding of transfusion reactions within this specific population.

The development and application of molecular methods for blood group genotyping in patients with thalassemia could provide more precise cross-matching and monitoring of transfusion responses. Genotyping offers a detailed understanding of blood group antigen profiles beyond the standard serological methods, potentially reducing the risk of alloimmunization and improving transfusion safety and outcomes. Understanding the distribution of ABO, Rhesus, and Kell genotypes in the Indonesian Thalassemia population and their association with hemolysis could inform clinical practice and guide policy changes toward more personalized transfusion strategies [11,12].

In addition, genotype-phenotype discordance can occur in blood group antigens where the genetic data does not align with phenotypic expression due to factors like genetic mutations or epigenetic regulation. Understanding such discordance is crucial as it may affect transfusion outcomes and necessitate more sophisticated diagnostic approaches [13]. Moreover, other genetic factors, such as human leucocyte antigens or nonhuman leucocyte antigens genes, may influence the immune response to transfusions and impact the risk of hemolysis and alloimmunization. These aspects are essential for a comprehensive understanding of transfusion complications and are potential areas for future research [6].

### Aim of the Study

The study aimed to analyze the association of blood group genotyping with the incidence of hemolysis in patients with thalassemia undergoing repeated transfusions at Banyumas Regional Hospital, a key referral center for thalassemia care in Indonesia. By identifying specific genotypes that predispose patients to hemolytic reactions, the study seeks to contribute to the development of tailored transfusion strategies that enhance patient care and reduce the risk of adverse outcomes.

## Methods

### Design

This study is a cross-sectional, observational analytic study designed to assess the association between blood group genotyping and the incidence of hemolysis in β-thalassemia major patients undergoing repeated transfusions. The study follows the STROBE (Strengthening the Reporting of Observational studies in Epidemiology). The completed STROBE checklist can be found in the Multimedia Appendix 1. STROBE guidelines are used for reporting observational studies cross-sectional design allows for a snapshot of data to identify potential associations between genotypes and hemolysis at a single point in time, it does not establish causality or capture changes over time [14]. This limitation highlights the need for future longitudinal studies to explore these relationships dynamically and over extended periods.

### Setting

The study was conducted at Banyumas Regional Hospital in Central Java, Indonesia, from February 1, 2024, to the present. Banyumas Regional Hospital is a referral center for thalassemia care in southern Central Java and surrounding areas, providing an integrated thalassemia service since 2009.

### Population and Research Participants

The participants of this study were patients with thalassemia who underwent repeated transfusions at Banyumas Hospital. Inclusion and exclusion criteria are summarized in Textbox 1.

**Inclusion and exclusion criteria for study participation.**

**Inclusion criteria**:

- Age 0 to less than 18 years
- Participants were diagnosed with β-thalassemia major based on history taking, clinical examination, and hemoglobin electrophoresis results. Received red blood cell transfusions at a minimum interval of 2-4 weeks.
- Participants had received transfusions of at least 20 units [15].
- Received packed red cell blood transfusions (store 7 days).

**Exclusion criteria**:

- Participants with a diagnosis of leukemia.
- Participants with a diagnosis of lymphoproliferative disease.
- Participants with a diagnosis of diabetes.
- Participants with a diagnosis of solid tumors.
- Participants have immunosuppression disorders.

Calculation of sample size estimated proportion (prevalence) in finite population [15]. An assumed prevalence of 8%, based on the study conducted by Kasraian et al [11]. The minimum required sample size was determined to be 90 participants, assuming a 95% CI and a margin of error of 5%. The prevalence estimate was derived from previous studies reporting hemolysis rates in similar patient populations. The final sample size included 134 patients to account for potential dropouts and to ensure adequate power to detect significant associations between blood group genotypes and hemolysis. The sampling method is simple random sampling.

### Data Collection and Measurement

Venous blood samples were taken as much as 6 cc in EDTA and without coagulant tubes, as well as urine samples. Genotyping examination using Allele-Specific polymerase chain reaction (PCR ASP) and phenotyping using immunoserology [12]. Follow-up gene sequencing to see blood group variants [13].

### Genotyping and Phenotyping Identification

#### Genotyping

Blood group genotyping for ABO, Rhesus, and Kell alleles was performed using PCR ASP. Primers were designed and validated to ensure specificity and efficient amplification (Multimedia Appendices 2 and 3 for further details). Design criteria included appropriate length, GC content, and melting temperature, with in silico verification using NCBI (National Center for Biotechnology Information) BLAST. Experimental validation involved PCR (polymerase chain reaction) optimization, amplicon size confirmation by gel electrophoresis, and sequencing for allele confirmation. Quantitative PCR was used where necessary, with standard curve and melt curve analysis to ensure reliable quantification [16].

#### Phenotyping

Immunoserological phenotyping was conducted using the hemagglutination technique. The gel cards used were from Bio-Rad Laboratories, Dia Med GmbH, Switzerland for the detection of anti-A, anti-B, anti-D, anti-C, anti-E, and anti-K antibodies. Forward grouping was performed by reacting blood cells with antisera, while reverse grouping was performed by reacting serum samples with control blood cells. All reagents and techniques were standardized, and external quality controls were implemented to ensure the accuracy of the phenotyping results. Plasma samples that exhibited a positive result with the ID-Daicel antibody screen underwent antibody identification using an extended commercial 11-cell identification panel (ID-DiaPanel) using LISS/Coombs.

### Identification of Hemolysis

Markers of hemolysis (haptoglobin, free hemoglobin, lactate dehydrogenase [LDH], bilirubin, and hemoglobinuria) were examined before and after transfusion [5,17]. Hemolysis was identified using the following cutoff values: plasma free hemoglobin>10 mg/dL, haptoglobin <25 mg/dL, LDH >300 U/L, indirect bilirubin>2 mg/dL, and urine hemoglobin >100 ng/mL. These cutoffs were used to assess the severity of hemolysis.

Alloimmunization, defined as the presence of alloantibodies against RBC antigens, will be identified using an indirect antiglobulin test. Positive indirect antiglobulin test results will prompt antibody identification (ID-DiaPanel) focusing on clinically significant antigens within the ABO, Rh, and Kell systems.

### Data Analysis

The data analysis for this study will involve a comprehensive approach to evaluate the association between blood group genotyping and the incidence of hemolysis in β-thalassemia major patients undergoing repeated transfusions. A descriptive analysis will be conducted for all measured variables, with outliers assessed using boxplots and subsequently excluded from further analyses. The primary measure will be the incidence of hemolysis, evaluated through laboratory markers such as haptoglobin, free hemoglobin, LDH, bilirubin, and hemoglobinuria, measured both before transfusion and 1 hour post transfusion. Bivariate analyses using chi-square tests will be used to analyze the effects of various factors on hemolysis and alloimmunization. Following this, logistic regression models will identify independent predictors of hemolysis while adjusting for potential confounders identified in preliminary analyses. The regression models will include variables found significant in the bivariate analysis, with *P* values of <.05 considered statistically significant. Key independent variables include blood group genotypes (ABO, Rhesus, and Kell), age, frequency of transfusions, duration of thalassemia diagnosis, underlying medical conditions, and iron chelation therapy status. The selection of these variables was guided by previous literature identifying factors associated with hemolysis and their clinical relevance. Our primary hypothesis posits that specific blood group genotypes are significantly associated with an increased incidence of hemolysis in patients with thalassemia with repeated blood transfusion. In addition, we hypothesize that clinical factors such as age, transfusion frequency, and durations will also significantly influence hemolysis incidence. Missing data will be managed using multiple imputation methods to minimize bias, and sensitivity analyses will compare results from complete-case analyses with those including imputed data to ensure robustness.

### Ethical Considerations

This study adheres to ethical standards for research involving human participants, as mandated by institutional guidelines and international regulations.

#### Human Subject Ethics Review Approvals

The study protocol has been approved by the Ethics Committee of the Faculty of Medicine, Public Health, and Nursing at Gadjah Mada University (approval number 1511/UN1/FK-KMK.3/S3KK.3/PJ/2023). This approval ensures that all research activities comply with ethical principles for conducting research involving human participants.

#### Informed Consent

Informed consent was obtained from all participants or their legal guardians before participation in the study. Participants were informed of their rights, including the ability to withdraw from the study at any time without any impact on their medical care. For secondary analyses using existing data, we confirm that the original consent covers secondary analysis without requiring additional consent.

#### Privacy and Confidentiality

All data collected during this study will be anonymized to protect participant identities. Personal identifiers will be removed from datasets, ensuring that individual participants cannot be identified from the data. Data will be stored securely and accessed only by authorized personnel involved in the study.

#### Compensation Details

Participants will not receive monetary compensation. Instead, they will be provided with the results of their blood group genotyping and phenotyping as part of their participation in the study. The compensation will be communicated clearly to participants during the consent process, ensuring transparency and fairness in the compensation process.

#### Identification of Participants in Images

No identifiable images of participants will be included in the article or Multimedia Appendices 1-3. If any images are deemed necessary for inclusion, a statement confirming that consent has been granted from identifiable individuals will be provided, along with relevant consent forms uploaded during resubmission.

## Results

Data collection for this study was funded in June 2024, with recruitment and sample collection conducted from July 2024 to January 2025. A total of 90 samples were collected, and clinical and laboratory data compilation has been completed. Data analyses are currently underway, and the first results are expected to be reported in May 2025.

**Figure 1:**
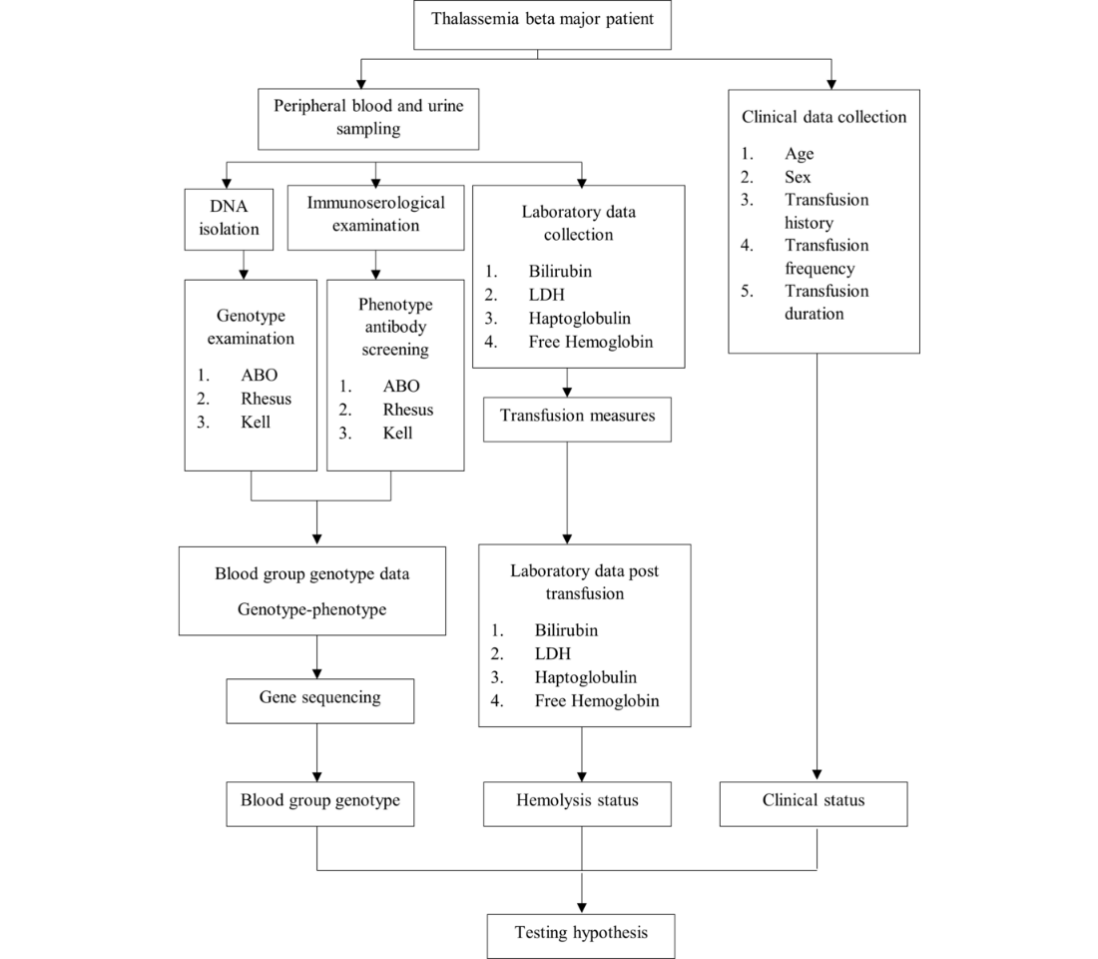
Flowchart of β-thalassemia major participants, eligible participants will be enrolled, and peripheral blood and urine samples will be collected before and after transfusion. Required clinical data will be collected from patients’ medical records. LDH: lactate dehydrogenase.

The flow diagram is outlined in Figure 1.

## Discussion

### Principal Findings

Our study generates baseline data for the Banyumas region in Indonesia, allowing for the development of targeted transfusion strategies tailored to the local population. These strategies contribute to the global understanding of thalassemia management by highlighting the importance of considering regional variations in blood group genetics. Thus, even with the limited sample size, the initial findings have the potential to inform clinical practice and policy changes within Indonesia, laying the groundwork for more personalized transfusion strategies and to global understanding of thalassemia in regions with limited data.

Preliminary findings suggest an association between certain blood group genotypes and higher hemolysis rates in patients with thalassemia receiving repeated transfusions. Though complete results are pending, initial data trends support our hypothesis that specific genotypes, especially within the ABO and Rh systems, correlate with increased hemolysis events, likely due to alloimmunization risks tied to blood group antigens [5].

Observed variations in markers such as LDH, bilirubin, and haptoglobin levels in particular genotypes strengthen the case for targeted monitoring. These trends suggest that some blood group genotypes may predispose individuals to hemolytic reactions, reinforcing the importance of considering genotype-matching in transfusion protocols for patients with thalassemia. While more data is needed for a conclusive analysis, these early insights indicate that genotype-specific risks should be addressed to enhance transfusion safety and efficacy [11].

Specifically, we anticipate that identifying specific blood group genotypes associated with hemolytic reactions will lead to more personalized transfusion strategies, ultimately improving patient care and outcomes [3]. This approach situates the study’s purpose within the broader context of enhancing patient safety and expanding academic knowledge in this critical area of transfusion medicine.

### Limitations

This study has identified several limitations. First, the study’s sample size of 90 patients may not adequately capture the full genetic and clinical diversity found in larger populations of β-thalassemia major patients in Indonesia or globally. Second, the single-center design of this study may introduce selection bias. This could limit the generalizability of the findings. Third, a cross-sectional study offers only a snapshot of data at a single point in time, failing to account for changes over time or establish causality between genotypes and the incidence of hemolysis. Furthermore, the absence of a cost-effectiveness analysis in this study limits our understanding of the economic implications of implementing genotype-based blood group testing in this population. Future research should address this limitation by conducting a formal cost-effectiveness analysis to evaluate the financial impact of routine genotyping in patients with thalassemia. Therefore, further investigation with larger populations, multicenter studies, and implementing a longitudinal design will be necessary to ensure generalizable findings and help establish causality and track changes over time.

Future research should investigate the long-term impacts and cost-effectiveness of blood group genotyping, and the genetic basis of alloimmunization in thalassemia. This information can inform policies to integrate genotyping into transfusion protocols, enhancing patient safety and quality of life.

### Dissemination Plan

The dissemination plan for this study on the role of blood group genotyping in predicting hemolysis among people with β-thalassemia major undergoing repeated transfusions involves multiple strategies to ensure broad impact and application in clinical practice. We will submit the primary results for publication in high-impact, peer-reviewed medical journals and present them at national and international conferences to engage with experts and foster collaborations. In addition, workshops and seminars will be conducted at local hospitals and universities to educate health care professionals about the study’s findings and their practical applications. Policy briefs and recommendations will be formulated for health care authorities and policy makers to promote the integration of blood group genotyping into standard transfusion protocols. Collaborating with patient advocacy groups will ensure that information is widely shared through newsletters, social media, and educational materials. Simplified educational materials will be distributed in hospitals, clinics, and support groups. The findings and resources will also be available on open-access platforms and internet-based forums to engage the global thalassemia and transfusion medicine community. This approach aims to inform clinical practice, guide policy development, and enhance patient education, ultimately advancing thalassemia care globally, with a focus on transitional countries.

### Conclusions

Our study highlights the complex interaction between blood group genotypes and hemolysis risk in patients with thalassemia undergoing repeated transfusions. Early findings suggest that certain genotypes, particularly in the ABO, Rhesus, and Kell blood group systems, may be associated with a higher incidence of hemolysis due to alloimmunization. This underlines the importance of exploring molecular genotyping to ensure better transfusion compatibility. Observing variations in hemolysis markers such as LDH, bilirubin, and haptoglobin in relation to specific blood genotypes, our research emphasizes the potential benefits of genotype-matched transfusions. By identifying genotype-specific responses, we aim to reduce hemolysis and improve patient outcomes in long-term transfusion therapy. Further research with a larger cohort will be essential to confirm these preliminary associations and develop targeted transfusion strategies that prioritize genotype compatibility. Ultimately, this study serves as a foundation for implementing genotype-informed transfusion protocols, promising improved care quality and safety for patients with thalassemia.

## Appendix

Multimedia Appendix 1 STROBE (Strengthening the Reporting of Observational studies in Epidemiology) statement—checklist of items that should be included in reports of observational studies.

Multimedia Appendix 2 Protocol for identification hemolysis.

Multimedia Appendix 3 Primers used in allele-specific polymerase chain reaction (PCR ASP) for ABO, Rh, and Kell blood group genotyping.

## Abbreviations

LDH: lactate dehydrogenase
NCBI: National Center for Biotechnology Information
PCR: polymerase chain reaction
PCR ASP: Allele-Specific polymerase chain reaction
RBC: red blood cell
STROBE: Strengthening the Reporting of Observational studies in Epidemiology
