# Supplementary material for: Using Blood Group Genotyping to Predict Hemolysis in Patients With β-Thalassemia Major With Frequent Transfusions: Protocol for a Cross-Sectional Study
Source: JMIR Res Protoc. 2025 May 30;14:e64379. doi: 10.2196/64379 (PMC12166315; doi:10.2196/64379)
Supplement: Multimedia Appendix 1 [file resprot_v14i1e64379_app1.docx]

STROBE Statement—checklist of items that should be included in reports of observational studies

|  | Item No. | Recommendation | Page  No. | Relevant text from manuscript |
| --- | --- | --- | --- | --- |
| **Title and abstract** | 1 | (*a*) Indicate the study’s design with a commonly used term in the title or the abstract | 1 | Analysis of Blood Group Genotyping as A Predictor of Hemolysis Incidence in Thalassemia Beta Major Patients with Repeated Transfusions: Protocol for an Observational Cross-Sectional Study |
|  |  | (*b*) Provide in the abstract an informative and balanced summary of what was done and what was found | 1 | In total, ninety people with thalassemia beta major were included in this study. Data analysis will start in June 2024. |
| Introduction | | | |  |
| Background/rationale | 2 | Explain the scientific background and rationale for the investigation being reported | 2 | Patients with thalassemia beta who require repeated blood transfusions are at risk of transfusion-related complications, such as excessive iron accumulation, alloimmunization, infection and transfusion reactions. |
| Objectives | 3 | State specific objectives, including any prespecified hypotheses | 2 | The study was designed to analyze the association of blood group genotyping with the incidence of hemolysis in thalassemia patients undergoing repeated transfusions. |
| Methods | | | |  |
| Study design | 4 | Present key elements of study design early in the paper | 4 | This study uses an observational analytic research design with a cross-sectional approach in which the data to be used comes from thalassemia patients who will undergo repeated transfusions at Banyumas Hospital. |
| Setting | 5 | Describe the setting, locations, and relevant dates, including periods of recruitment, exposure, follow-up, and data collection | 5 | Setting and figure 1 |
| Participants | 6 | (*a*) Give the eligibility criteria, and the sources and methods of selection of participants | 6 | The population of this study were Thalassemia patients who underwent repeated transfusions at Banyumas Hospital. Inclusion and Exclusion criteria are summarized in Textbox 1. |
| Variables | 7 | Clearly define all outcomes, exposures, predictors, potential confounders, and effect modifiers. Give diagnostic criteria, if applicable | 7 | Markers of hemolysis (haptoglobulin, free hemoglobin, LDH, bilirubin, hemoglobinurin) were examined using Cobas C113, ELISA and urinalysis early before transfusion and one hour after transfusion. The incidence of hemolysis was determined based on several criteria: haptoglobulin, free hemoglobin, LDH, bilirubin, hemoglobinurin. |
| Data sources/ measurement | 8* | For each variable of interest, give sources of data and details of methods of assessment (measurement). Describe comparability of assessment methods if there is more than one group |  | Antibody screening and phenotyping serological examination of antibody screening and blood groups ABO, Rhesus and Kell samples used in this study in the form of blood using immunoserology.  Genotyping blood groups ABO Rhesus and Kell using PCR ASP  Hemolysis marker using serum blood and measurement with ELISA Methods |
| Bias | 9 | Describe any efforts to address potential sources of bias | 7 | We have inclusion and exclusion criteria for the sources |
| Study size | 10 | Explain how the study size was arrived at |  | Based on the formula and the prevalence assumption, the minimum sample size needed is ninety subjects. The sampling method is simple random sampling. |

Continued on next page

| Quantitative variables | 11 | Explain how quantitative variables were handled in the analyses. If applicable, describe which groupings were chosen and why | 7 | Descriptive analysis for all variable measured will be presented. There will also be bivariate analysis to analyze the effects of variables on the incidence of hemolysis and alloimmunization using chi-square. Following logistic regression test to examine the association between age, gender, genotype, transfusion history, transfusion duration and transfusion frequency with the incidence of hemolysis and alloimmunization. |
| --- | --- | --- | --- | --- |
| Statistical methods | 12 | (*a*) Describe all statistical methods, including those used to control for confounding | 7 | Descriptive analysis for all variable measured will be presented. There will also be bivariate analysis to analyze the effects of variables on the incidence of hemolysis and alloimmunization using chi-square. Following logistic regression test to examine the association between age, gender, genotype, transfusion history, transfusion duration and transfusion frequency with the incidence of hemolysis and alloimmunization. |
|  |  | (*b*) Describe any methods used to examine subgroups and interactions | 7 | Descriptive analysis for all variable measured will be presented. There will also be bivariate analysis to analyze the effects of variables on the incidence of hemolysis and alloimmunization using chi-square. Following logistic regression test to examine the association between age, gender, genotype, transfusion history, transfusion duration and transfusion frequency with the incidence of hemolysis and alloimmunization. |
|  |  | (*c*) Explain how missing data were addressed | 7 | We analyze only complete cases; missing data would be dropped instantly. |
|  |  | (*d*) If applicable, describe analytical methods taking account of sampling strategy | 6 | The sampling method is simple random sampling. |
|  |  | (*e*) Describe any sensitivity analyses | 7 | We analyze only complete cases; missing data would be dropped instantly. |
| Results | | | | |
| Participants | 13* | (a) Report numbers of individuals at each stage of study—eg numbers potentially eligible, examined for eligibility, confirmed eligible, included in the study, completing follow-up, and analysed | 7 | Will be reported once data analyses has been undertaken |
|  |  | (b) Give reasons for non-participation at each stage | 7 | Will be reported once data analyses has been undertaken |
|  |  | (c) Consider use of a flow diagram | 5 | The study procedure is outlined in Figure 1 |
| Descriptive data | 14* | (a) Give characteristics of study participants (eg demographic, clinical, social) and information on exposures and potential confounders | 7 | Will be reported once data analyses has been undertaken |
|  |  | (b) Indicate number of participants with missing data for each variable of interest | 7 | Will be reported once data analyses has been undertaken |
| Main results | 16 | (*a*) Give unadjusted estimates and, if applicable, confounder-adjusted estimates and their precision (eg, 95% confidence interval). Make clear which confounders were adjusted for and why they were included | 7 | Will be reported once data analyses has been undertaken |
|  |  | (*b*) Report category boundaries when continuous variables were categorized | 7 | Will be reported once data analyses has been undertaken |
|  |  | (*c*) If relevant, consider translating estimates of relative risk into absolute risk for a meaningful time period | 7 | Will be reported once data analyses has been undertaken |

Continued on next page

| Other analyses | 17 | Report other analyses done—eg analyses of subgroups and interactions, and sensitivity analyses | 7 | Will be reported once data analyses has been undertaken |
| --- | --- | --- | --- | --- |
| Discussion | | | | |
| Key results | 18 | Summarise key results with reference to study objectives | 8 | Principal Results |
| Limitations | 19 | Discuss limitations of the study, taking into account sources of potential bias or imprecision. Discuss both direction and magnitude of any potential bias | 8 | Several limitations are identified in this study. |
| Interpretation | 20 | Give a cautious overall interpretation of results considering objectives, limitations, multiplicity of analyses, results from similar studies, and other relevant evidence | 8 | Will be reported once data analyses has been undertaken |
| Generalisability | 21 | Discuss the generalisability (external validity) of the study results | 8 | Will be reported once data analyses has been undertaken |
| Other information | |  | | |
| Funding | 22 | Give the source of funding and the role of the funders for the present study and, if applicable, for the original study on which the present article is based | 9 | Acknowledgements |

*Give information separately for cases and controls in case-control studies and, if applicable, for exposed and unexposed groups in cohort and cross-sectional studies.

**Note:** An Explanation and Elaboration article discusses each checklist item and gives methodological background and published examples of transparent reporting. The STROBE checklist is best used in conjunction with this article (freely available on the Web sites of PLoS Medicine at http://www.plosmedicine.org/, Annals of Internal Medicine at http://www.annals.org/, and Epidemiology at http://www.epidem.com/). Information on the STROBE Initiative is available at www.strobe-statement.org.
