# Supplementary material for: Using Blood Group Genotyping to Predict Hemolysis in Patients With β-Thalassemia Major With Frequent Transfusions: Protocol for a Cross-Sectional Study
Source: JMIR Res Protoc. 2025 May 30;14:e64379. doi: 10.2196/64379 (PMC12166315; doi:10.2196/64379)
Supplement: Multimedia Appendix 2 [file resprot_v14i1e64379_app2.pdf]

Comment 3: References are needed for the standard methods for identification of hemolysis and the specific equipment and protocols followed.

Protocol for identification of hemolysis

Hemolysis events are determined using several criteria and measuring instruments as follows:

1. LDH Measurement

   Required equipment: Syringe, 70% alcohol swab, tourniquet, plaster, and 3 cc plain tube.

   Procedure:

   a. Draw 3 cc of blood from the median cubital vein.
   b. Place the sample in a plain tube.
   c. Centrifuge at 3000 rpm for 10 minutes.
   d. Use the resulting serum to test LDH with a Cobas C311 chemistry analyzer.

2. Bilirubin Measurement

   Required equipment: Syringe, 70% alcohol swab, tourniquet, plaster, and 3 cc plain tube.

   Procedure:

   a. Draw 3 cc of blood from the median cubital vein.
   b. Place the sample in a plain tube.
   c. Centrifuge at 3000 rpm for 10 minutes.
   d. Test the resulting serum for indirect bilirubin using a Cobas C311 chemistry analyzer.

3. Haptoglobin Measurement

   Required equipment: Syringe, 70% alcohol swab, tourniquet, plaster, and 3 cc EDTA tube.

   Procedure using ELISA method:

   a. Prepare reagents, samples, and standards, designating wells in the plate for standard, blank, and sample.
   b. Add 100 µL of standard and sample to designated wells, cover with a sealer, and incubate at 37˚C for 90 minutes.
   c. After incubation, discard the liquid from each well without washing.

d. Add 100 µL of Biotinylated Detection Ab solution to each well and incubate at 37˚C for 60 minutes.
e. Discard the liquid and add 350 µL of wash buffer to each well, let it sit for 1 minute, aspirate, and dry. Repeat this washing step three times.
f. Add 100 µL of HRP conjugate solution to each well, cover with a new sealer, and incubate at 37˚C for 30 minutes.
g. Discard the liquid again, and repeat the wash procedure five times.
h. Add 90 µL of substrate reagent to each well, cover, and incubate at 37˚C for 15 minutes.
i. Add 50 µL of stop solution to each well.
j. Read the Optical Density (OD) at 450 nm using a microplate reader.

4. Free Hemoglobin Measurement

   Required equipment: Syringe, 70% alcohol swab, tourniquet, plaster, and 3 cc EDTA tube.

   Procedure (similar ELISA method as for Haptoglobin):

   Follow a similar ELISA procedure as outlined for haptoglobin, including incubations, washing steps, and Optical Density (OD) reading at 450 nm.
5. Hemoglobinuria Measurement

   Required equipment: Urine collection tube.

   Procedure using ELISA method:

   Follow similar ELISA steps as outlined for haptoglobin, including incubation, washing, and Optical Density (OD) reading at 450 nm.
