# Supplementary material for: Using Blood Group Genotyping to Predict Hemolysis in Patients With β-Thalassemia Major With Frequent Transfusions: Protocol for a Cross-Sectional Study
Source: JMIR Res Protoc. 2025 May 30;14:e64379. doi: 10.2196/64379 (PMC12166315; doi:10.2196/64379)
Supplement: Multimedia Appendix 3 [file resprot_v14i1e64379_app3.docx]

Table. ABO, Rhesus, and Kell Blood Group Primers (Hojjati et al., 2011; Muro et al., 2012; Khosroshahi et al., 2019)

| Blood Groups | Primer | Nukleotida 5’🡪 3’ | Allele Spesific | Size (bp) |
| --- | --- | --- | --- | --- |
| ABO | Forward | GATGTCCTCGTGGTAC | O | 74 |
|  | Reverse | CTCGTTGAGGATGTCGATGTTG |  |  |
|  | Forward | GGAAGGATGTCCTCGTGGTGA | AB | 79 |
|  | Reverse | CTCGTTGAGGATGTCGATGTTG |  |  |
|  | Forward | GACGAGGGCGATTTCTACTACA | B | 107 |
|  | Reverse | TTGGCCTGGTCGACCATCATG |  |  |
|  | Forward | TCTACTACCTGGGGGG | AO | 94 |
|  | Reverse | TTGGCCTGGTCGACCATCATG |  |  |
| Rhesus | Forward | ACGATACCCAGTTTGTCT | D | 600 |
|  | Reverse | TGACCCTGAGATGGCTGT |  |  |
|  | Forward | CGCTGCCTGCCCCTCTGC | C | 118 |
|  | Reverse | TTGATAGGATGCCACGAGCC |  |  |
|  | Forward | CTTGGGCTTCCTCACCTCAAA | c | 107 |
|  | Reverse | AAGCCGTCCAGCAGGATTGC |  |  |
|  | Forward | TGGCCACGTGTCAACTCTC | E | 143 |
|  | Reverse | CATGCTGATCTTCCTTTGGG |  |  |
|  | Forward | TGGCCACGTGTCAACTCTG | e | 143 |
|  | Reverse | CATGCTGATCTTCCTTTGGG |  |  |
| Kell | Forward | GACTTCCTTAAACTTTAACCGCAT | K1 | 609 |
|  | Reverse | CGCCAGTGCATCCCTCACC |  |  |
|  | Forward | GGACTTCCTTAAACTTTAACCGCAC | K2 | 535 |
|  | Reverse | CGCCAGTGCATCCCTCACC |  |  |
